# Digitised remote delivery of simulation in psychiatry during the pandemic and for the future

**DOI:** 10.1192/j.eurpsy.2022.523

**Published:** 2022-09-01

**Authors:** J. Mudunkotuwe, V. Mannali, J. Henry, J. Clift, P. Strickland

**Affiliations:** 1 Surrey and Borders NHS Foundation Trust, Medical Education, Epsom, United Kingdom; 2 Xenadu Virtual environments, It Consultancy, hertfordshire, United Kingdom

**Keywords:** virtual reality, pandemic, remote education, simulation

## Abstract

**Introduction:**

Surrey and Borders NHS Foundation Trust’s AVATr (Augmented Virtual-reality Avatar in Training) is a unique ground-breaking Virtual Patient simulation system, which uses the Xenodu platform to train learners in essential clinical and complex communication skills. Over 30 patient scenarios have been developed after identifying learner-specific development needs, including exploration of overt psychosis, assessment of capacity, sharing bad news, and neglect in care home residents.

During the session, the trainee is projected on to a large screen, using a camera and video special effects, which results in a life-like interaction with the Virtual Patient. Trainees can view themselves interacting with the Virtual Patient in real-time, from a unique ’out-of-body’ perspective, immersed in a customdesigned interactive virtual environment. This is different to a first-person perspective used in virtual or augmented-reality systems in several clinical specialties. During the COVID-19 pandemic, we evolved the AVATr model to remote or hybrid sessions, where simulations were digitally enhanced, and have been run through Microsoft Teams. The simulation facilitator is connected to a multi-user video call, enabling the Virtual Patient to be projected as an attendee using Microsoft Teams.

**Objectives:**

To evaluate the feedback from Doctors in training taking part on the education sessions.

**Methods:**

We collected qualitiative and quanttaive infromation from participants after the teaching session.

**Results:**

We received strongly positive reults in all parameters measured. the presenters will show a detailed breakdoen in the session.

**Conclusions:**

The digitalised delivery of the virtual patient simulation, has been pivotal in limiting interruptions to communication skills training in mental health.

**Disclosure:**

The NHS trust has co produced the simulation platform with a private software firm Xenadu Virtual Environments

